# Using Both Sides of Your Brain: The Case for Rapid Interhemispheric Switching

**DOI:** 10.1371/journal.pbio.0060269

**Published:** 2008-10-28

**Authors:** Marc F Schmidt

## Abstract

Individual brain hemispheres are often specialized for specific aspects of a behavior. How both sides of the brain coordinate their output to produce a perfectly seamless behavior is not known. Songbirds appear to achieve this by rapidly switching back and forth between hemispheres.

The vertebrate brain is symmetrically organized, with two identical-looking cerebral hemispheres and a brainstem that contains identically named nuclei on both sides of the midline. However tempting it is to think of each brain half as a mirror image of its contralateral counterpart, this is an incorrect oversimplification. Careful anatomical comparison of select anatomical structures and their connections reveals asymmetries across sides [1]. These asymmetries are not limited to anatomical features but also include extensive functional differences between cerebral hemispheres [2]. The existence of asymmetries implies that the two hemispheres are not just two massively redundant networks, but rather functionally specialized entities that work synergistically to coordinate the behavior of the organism.

## Hemispheric Specialization as a General Feature of Brain Systems

Hemispheric specialization, or lateralization as it is often referred to, was originally thought to be a unique human characteristic but appears to be a general property among vertebrate brains. There are many examples of hemispheric lateralization, such as the specialization of the right hemisphere to process and store visual inputs that are important for imprinting in chicks [3], and the different auditory processing characteristics in the left and right auditory forebrain of songbirds [4,5]. There are also many species of amphibians, reptiles, fish, and birds that show functional lateralization at the periphery that suggests hemispheric specialization [6]. A striking example is the way in which many migratory birds sense the direction of the magnetic field using only their right eye (magnetoperception is detected by specialized ganglion cells in the retina) [7].

It was originally believed that specific behaviors were entirely lateralized to a single hemisphere [8]. The emerging view, however, is that individual hemispheres are not necessarily dedicated to a single behavior but are instead specialized for specific features of those behaviors [9]. In the context of language, for example, both hemispheres are involved in some aspect(s) of speech processing and production, even though the left hemisphere might appear more dominant. The left hemisphere is, for example, more specialized for lexical and syntactical aspects of language, while the right hemisphere is more sensitive to emotional features of speech [10]. There is supportive evidence that properties of the auditory cortex parallel these specializations. The left auditory cortex, for example, is more sensitive to fast temporal features of sound, which are necessary for phonemic-level speech processing. In contrast, the right auditory cortex is sensitive to the slow rhythmic patterns in sounds that are associated with prosody, which is the rhythm, stress, and intonation of speech [11]. Even at the level of speech production, a behavior originally thought to be controlled exclusively by the left hemisphere, there is now clear evidence that both hemispheres participate in the phonation process [12].

## Interhemispheric Switching Can Occur under a Wide Range of Conditions and Time Scales

Many behaviors require the recruitment of specializations from each hemisphere. In the case of language, for example, syntax and prosody need to be combined to produce coherent speech patterns. Given the known hemispheric specializations for language, this combination requires the coordinated engagement of both hemispheres. This engagement might be simultaneous, i.e., both hemispheres are active at the same time, or it might conceivably occur in alternation, i.e., hemispheric control switches from one hemisphere to the other. Although the concept of hemispheric switching during the production of a single goal-directed behavior might not seem obvious, switching in hemispheric activation has been observed in a number of animals, including humans.

Hemispheric switching can be observed under a variety of conditions and on multiple time scales. At the slow end of the spectrum, switching back and forth between hemispheres can be observed during sleep in many aquatic mammals and birds [13]. Sleep-like activity (as measured by EEG recording) will occur in a single hemisphere for several minutes at a time before switching over to the contralateral hemisphere [14]. At slightly faster rates, interhemispheric switching can be observed during eye movement in animals that can independently control each eye, such as chameleons and sandland fish. These animals never generate saccades in both eyes at the same time, but instead generate a run of saccades in one eye before switching to the other eye after 10 to 20 seconds. Because the visual pathways in these animals are entirely crossed, the pattern of eye movements implies hemispheric switching at that same rate [15]. At the fast end of the spectrum, interhemispheric switching can occur at a rate of about 1 Hz during specific perceptual rivalry tasks in humans [16]. In the case of binocular rivalry, where different visual stimuli (horizontal lines in one eye and vertical lines in the other) are presented one to each eye simultaneously, subjects alternate between perceiving either vertical or horizontal lines but rarely perceive both. Use of transmagnetic stimulation (a noninvasive technique akin to electrical stimulation) to perturb neural activity has been used to show that interhemispheric switching is synchronous with these observed perceptual switches [17]. Interestingly, the rate of interhemispheric switching during these tasks is not fixed because it can vary significantly with mood shifts and is much slower in subjects with manic depression [18].

Only a few examples of interhemispheric switching have been demonstrated to date. Nevertheless, the range of conditions over which such switching can be observed suggests that it might be a general mechanism of brain function in bilaterally organized brain systems. While the adaptive advantage of switching between hemispheres might be apparent in sleeping animals—because it allows animals the possibility of having one hemisphere in an awake state at all times—the role that such switching plays in motor production or perception is not known. In a new study from Hahnloser's group in the current issue of *PLoS Biology,* Wang et al. provide evidence, during song production in birds, for the fastest rate of interhemispheric switching yet described [19]. Importantly, because song production is controlled by a well-defined neural system and can be quantified with exquisite precision, the avian song system might prove to be an ideal model for providing insight into the functional implication of interhemispheric switching.

## Song Production in Birds: A Highly Asymmetric Behavior

**Figure 1:**
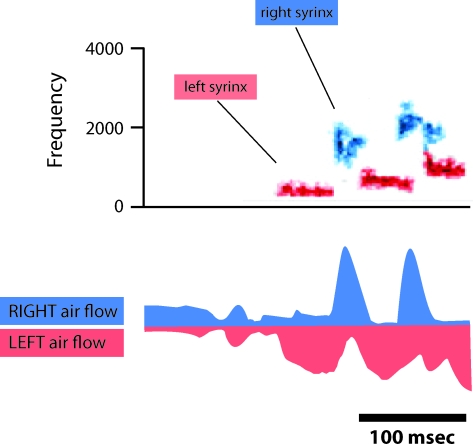
Songbirds Can Switch Rapidly between Sides When They Sing Many songbirds will use both their left and right syrinx to produce song. In some cases, as illustrated here by the brown-headed cowbird, they can switch rapidly from producing sound in the left syrinx to producing sound in the right syrinx. In this example, a cluster of five short song elements is produced within a very short period of about 200 ms. The contribution of each syringeal side can be measured by implanting a small heated microbead thermistor in each primary bronchus. These measure the rate of airflow through each side of the syrinx. The bottom portion of the figure shows the airflow through each bronchus with airflow from the right side in blue and left side in red. Note that left airflow has been flipped upside down to better compare with the right side. From these measurements, one can infer the syringeal source of each song element. As shown in the sonogram, cowbirds rapidly alternate between producing a note with the left (red) and the right (blue) sides of the syrinx. (Based on recordings performed in the cowbird by Rod Suthers)

Song production in passerine birds is one of the most striking and best characterized asymmetric behaviors at the peripheral level [20]. Each bird has a bipartite vocal organ, known as a syrinx, that is divided into left and right halves that each contain an independent membranous sound source [21]. The vocal output that one hears from a bird's beak is therefore the sum of the sounds generated from each “sound box.” In the majority of species studied, individual song syllables can be produced using a variety of different strategies. In some cases, sound can be generated in both syringeal halves at the same time, while in other cases, sound can be produced in one side at a time with air flow actively blocked in the non–sound producing syringeal half [22]. The strategies used to produce a given syllable can vary even within the same song, and there are many cases where alternations between sides can occur multiple times during the production of a single syllable (Figure 1). Switching between sides is so perfectly synchronized that the acoustic output is completely smooth across these transitions. It has been suggested that the ability of birds to use each syringeal half as a separately controlled “sound box” might be a strategy to increase the complexity of their songs [23].

**Figure 2:**
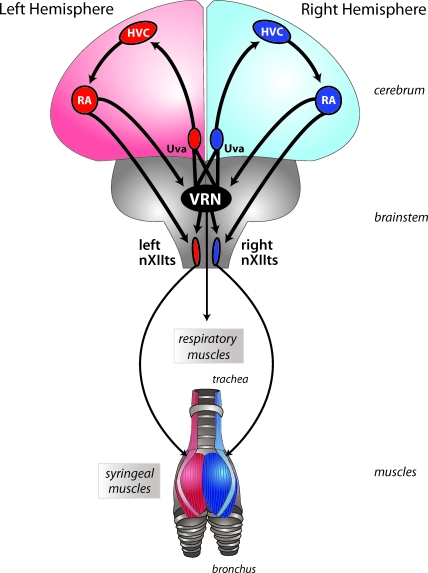
Bilateral Organization of the Song Motor Control System This schematic is a simplified representation of the motor portion of the avian song system emphasizing its bilateral organization. Sound is produced in a bipartite vocal organ known as the syrinx. The syringeal muscles that make up each half of the vocal organ are innervated by motoneurons in the ipsilateral nXIIts. This nucleus receives its own motor commands from HVC and RA in the ipsilateral forebrain. Therefore, motor commands generated in the left hemisphere (highlighted in red) end up activating muscles on the left half of the syrinx, while those on the right side (highlighted in blue) activate syringeal muscles on the right side. Motor commands from each hemisphere are also sent to brainstem nuclei that form part of the bilaterally organized vocal-respiratory network (VRN), which is highly interconnected across the midline. The VRN sends motor commands to the muscles of respiration. This network also sends major projections back to the forebrain nucleus HVC via the intermediary of the thalamic relay nucleus uvaeformis (Uva). These bottom-up projections are thought to synchronize activity in both hemispheres and might play a role in the rapid hemispheric switching described by Wang et al.

In songbirds, each hemisphere contains a discrete set of brain structures that control song production in adult birds [24]. Figure 2 is a schematic of the main descending motor pathway showing the connection between the forebrain nuclei HVC (used as a proper name) and RA (robust nucleus of the arcopallium) and the two major projections of RA to the brainstem. One of these projections goes directly to the ipsilateral brainstem's hypoglossal nucleus (nXIIts, the tracheosyringeal part of the hypoglossal nucleus), a motor output structure that exclusively innervates the ipsilateral syringeal muscles. The other major projection from RA innervates a series of highly interconnected vocal-respiratory nuclei in the ipsilateral brainstem, several of which send projections to respiratory motoneurons in the spinal cord. These nuclei are highly interconnected across the midline and can therefore be thought of as a bilaterally organized vocal-respiratory network (VRN).

The hypoglossal nuclei (nXIIts) that innervate the syringeal muscles do not connect across the midline, and muscles on each half of the syrinx therefore receive separate sets of motor commands from each ipsilateral motor pathway. Because song involves the simultaneous activation of muscles on both halves of the syrinx, it is critical that syringeal output be precisely synchronized and coordinated during song production. Songbirds, however, do not have direct projections between any of the cerebral song control nuclei in each hemisphere (birds do not have a corpus callosum), and so coordination must occur through a mechanism other than direct hemispheric connectivity. Because the brainstem VRN provides, via its bilateral projections back up to HVC (Figure 2), the major way in which forebrain song nuclei are functionally connected, it is well positioned to serve a key function in synchronizing song-related motor activity in each hemisphere [25]. Given the new results from Wang et al. [19], these projections might well also play a critical role in switching between hemispheres.

## Rapid Interhemispheric Switching Occurs during Precise Transitions in the Song

One approach to test whether a behavior is being driven by the rapid alternation of motor commands sent by each hemisphere is to briefly stimulate the circuits responsible for producing the behavior and ask whether the ability of such perturbation to alter the behavior is dependent on the specific hemisphere that is stimulated. In songbirds, it has been shown that brief electrical stimuli (a few short biphasic pulses at relatively low current levels) delivered directly to HVC, RA, or the VRN can interrupt the temporal structure of the ongoing song [26]. In zebra finches, whose song is made up of a stereotyped sequence of syllables (known as a motif) that is repeated multiple times, this interruption is observed as a truncation of the ongoing syllable (with a delay of approximately 70 ms) followed either by the termination of the song or the start of a new motif [26].

Using this technique, Wang and colleagues implanted electrodes in HVC of each hemisphere and found that stimulating HVC in one hemisphere caused song disruptions only when these short stimuli were delivered during certain portions of the motif. Astonishingly, the authors observed that stimulation of the contralateral HVC interrupted song at precisely the time when stimulation in the other HVC had no effect on song. In other words, left hemisphere stimulation disrupted song during periods when the right hemisphere stimulation was ineffective and vice versa. This pattern of left/right hemisphere switching could occur as often as three to four times during the production of a single 200 ms syllable. When the authors increased the amount of current, this effect disappeared and stimuli were effective at perturbing song temporal structure throughout the whole motif, suggesting that the threshold for perturbing syllable sequencing within a motif is what switches rapidly between hemispheres. The change in perturbation threshold in a given hemisphere does not necessarily imply that the “nondominant” hemisphere is inactive. Multi-unit neural recordings from HVC during singing in the same species [27] show clear premotor activity in each hemisphere during the entirety of the motif. The ability of electrical stimulation to perturb activity in each hemisphere in rapid alternation therefore reflects changes that are subtler than simple all-or-none changes in premotor activity.

Given the evidence for alternation between the left and right half of the syrinx, a critical question is whether the timing of hemispheric switching is synchronized to the switching observed in the syrinx. The authors did not directly measure syringeal dynamics, but they did compare switching patterns with the major acoustic transitions in the song. They did not find any obvious relationship between hemispheric switching and transitions in the song (e.g., syllable onset or offset) or acoustic feature transitions (e.g., pitch or amplitude). While this approach provides a reasonable initial characterization, extrapolating syringeal switching patterns from the acoustic signal is likely to be inaccurate. Future experiments combining HVC stimulation with syringeal muscle recordings or airflow measurements, parameters that are typically used to measure syringeal switching [28], are therefore necessary to unequivocally reveal any relationship between central (hemisphere) and peripheral (syrinx) switching. An additional direction that might be explored to link interhemispheric switching with song output is to compare interhemispheric switching patterns in birds that have been tutored with, and therefore learned, the same exact song, a process that is possible under specific tutoring paradigms [29]. Having birds sing identical songs would allow analysis across birds of the general types of song transitions that lead to interhemispheric switching.

## The Switching Mechanism: The Brainstem as a Possible Key Player

When hemispheres contribute differentially to behaviors that recruit muscles bilaterally, such as for bimanual movement or vocal production, it is sometimes assumed that hemispheric coordination is achieved exclusively by the corpus callosum, a massive fiber bundle that connects the left and right cerebral hemispheres. This commissural system is evolutionarily recent and is only observed in placental mammals, yet hemispheric switching is observed in animals such as fish and birds that do not have a corpus callosum. A possible neural substrate for hemispheric coordination and switching might therefore include brainstem neuromodulatory systems. These are interconnected across the midline and project diffusely throughout the hemispheres, and could therefore differentially influence each hemisphere. Consistent with this view, a recent study has shown that cortical release of the neurotransmitter acetylcholine is lateralized during asymmetric sleep in fur seals [30]. While this mechanism might be well suited for hemispheric switching during sleep, the generally slow time course of action of neuromodulators makes this mechanism somewhat less attractive for rapid hemispheric switching.

For fast switching, alternative mechanisms might include bilaterally coupled networks in the brainstem that directly “drive” each hemisphere. This is plausible in the song system given known projections from the brainstem back up to the forebrain via the intermediary of the thalamus, and the direct influence that these brainstem areas have on neural activity in forebrain song control nuclei [31]. Interestingly, a similar relationship between the brainstem and forebrain has been demonstrated in other motor control systems such as the primate oculomotor system [32]. Further supportive evidence for a role of the brainstem in hemispheric switching comes from findings that switching during binocular rivalry [17] can occur in the absence of a corpus callosum [33] and is modulated by serotonin receptor subtypes that are located largely in the brainstem [34]. For song production, the brainstem VRN is well placed to play a central role in hemispheric switching, but it is unlikely that it acts as a simple oscillating circuit, given that Wang and colleagues never observed any periodicity in the pattern of hemispheric switching. In fact, they often observed a wide range of switching intervals (from 4 ms to 150 ms) within a single song motif. An important direction for future work will be to identify the signal(s) responsible for the transitions that underlie hemispheric switching. In the song system, the VRN and the inputs it receives from RA are well placed to show spiking patterns that might be predictive of switching times.

## Functional Implications

One of the primary unanswered questions is why brain systems have evolved to switch rapidly between hemispheres. One possibility is that interhemispheric switching is part of a strategy to optimize processing power within each hemisphere while keeping both sides synchronized. Similar strategies are certainly used computationally in multicore processors. In songbirds, it is certainly clear from lesion studies that normal adult birds require both hemispheres to produce song [35]. The question of how the elements of song production are divided between hemispheres, however, is unclear. Wang and colleagues failed to find any relationship between switching times and features of song acoustics such as syllable onsets, yet they observed a wide range of switching time intervals. Presumably these intervals are not arbitrarily determined but reflect some aspect of song production, such as switching between sides of the syrinx. It is also important to note that the bilateral nature of the song system appears to have a learned component because unilateral lesions of RA in juvenile birds causes these birds to sing approximately normal songs as adults [35]. This suggests that the song system can compensate for whatever functional advantages are conferred by bilateral processing. An intriguing question for these “unihemispheric” birds is thus how this compensation occurs. For example, do unilateral birds also show switching at the level of the syrinx? If so, how is such switching achieved?

More generally, the purpose of interhemispheric switching may become clearer as more data are collected through the paradigm developed by the Wang lab, and similar experiments are performed in other motor systems. Functional specialization is found throughout the nervous system but requires communication among many different areas of the brain to result in cohesive activity. Interhemispheric switching provides a compelling example, and may very well elucidate more general principles of brain function and the production of temporally complex behaviors.

## Abbreviations

nXIIts: tracheosyringeal part of the hypoglossal nucleus
RA: robust nucleus of the arcopallium
VRN: vocal-respiratory network
